# Resolvin D1 Protects Podocytes in Adriamycin-Induced Nephropathy through Modulation of 14-3-3β Acetylation

**DOI:** 10.1371/journal.pone.0067471

**Published:** 2013-06-28

**Authors:** Xueming Zhang, Xinli Qu, Yu Bo Yang Sun, Georgina Caruana, John F. Bertram, David J. Nikolic-Paterson, Jinhua Li

**Affiliations:** 1 Department of Anatomy and Developmental Biology, Monash University, Wellington Road, Clayton, Victoria, Australia; 2 Baotou Medical College, Inner Mongolia University of Science and Technology, Baotou, People’s Republic of China; 3 Department of Medicine, Monash University, Clayton, Victoria, Australia; UAE University, Faculty of Medicine & Health Sciences, United Arab Emirates

## Abstract

Resolvin D1 (RvD1) is a lipid-derived mediator generated during the resolution inflammation. While the immunoresolvent effects of Resolvins have been extensively studied in leukocytes, actions of Resolvins on intrinsic kidney cells have received little attention. The podocyte plays a central role in glomerular function, and podocyte damage can lead to proteinuria and glomerulosclerosis. This study examined whether RvD1 has renoprotective effects upon podocytes. We investigated a mouse model of adriamycin (ADR) nephropathy featuring rapid induction of podocyte damage and proteinuria followed by glomerulosclerosis. We identified a progressive loss of synaptopodin expression over a 28 day time-course of ADR nephropathy which was associated with increased acetylation of 14-3-3β and reduced synaptopodin phosphorylation. Groups of mice were given once daily RvD1 treatment (4 ng/g body weight/day) starting either 30 min (early treatment) or 14 days (late treatment) after ADR injection and continued until mice were killed on day 28. Early, but not late, RvD1 treatment attenuated ADR-induced proteinuria, glomerulosclerosis and tubulointerstitial fibrosis, modified macrophages from an M1 to M2 phenotype. Early RvD1 treatment prevented the down-regulation of synaptopodin expression and changes in 14-3-3β acetylation and synaptopodin phosphorylation. In a podocyte cell line, RvD1 was shown to prevent rapid TNF-α-induced down-regulation of synaptopodin expression. In transfection studies, TNF-α-induced a decrease in synaptopodin phosphorylation and an increase in acetylation of 14-3-3β, resulting in disassociation between 14-3-3β and synaptopodin. RvD1 prevented TNF-α induced post-translational modification of synaptopodin and 14-3-3β proteins, and maintained the synaptopodin/14-3-3β interaction. Furthermore, replacement of lysine K51, or K117+K122 in 14-3-3β with glutamine, to mimic lysine acetylation, significantly reduced the interaction between 14-3-3β and synaptopodin. In conclusion, our studies provide the first evidence that RvD1 can protect against podocyte damage by preventing down-regulation of synaptopodin through inhibition of 14-3-3β/synaptopodin dissociation. RvD1 treatment may have potential application in the treatment of chronic kidney disease.

## Introduction

Resolvin D1 (RvD1) is a lipid mediator biosynthesized from docosahexaenoic acid during the resolution of inflammation [1]. RvD1 limits neutrophil infiltration in murine peritonitis [1], blocks transendothelial migration of human leukocytes [2], and enhances macrophage phagocytosis of zymosan and apoptotic polymorphonuclear leukocytes [3]. 17(R)-Resolvin D1 (17(R)-RvD1), an aspirin-triggered epimer of RvD1 [2], reduces leukocyte infiltration in a mouse model of peritonitis with equal potency to that of RvD1. Compared with RvD1, 17(R)-RvD1 resists rapid inactivation by eicosanoid oxidoreductases [2]. Both RvD1 and 17(R)-RvD1 modulate allergic airway response and promote macrophage clearance of allergens from the airways in an allergic mouse model [4]. Taken together, RvD1 and 17(R)-RvD1 demonstrate potent resolution of inflammation [5].

Podocytes are terminally differentiated cells of the glomerulus which make a major contribution to the glomerular filtration barrier so that albumin and larger proteins are retained in the blood. In addition, podocyte damage or loss can result in the development of glomerulosclerosis and the progression of glomerular disease to end-stage renal failure [6]. The maintenance of normal podocyte structure and glomerular filtration barrier function relies upon a highly dynamic actin cytoskeleton which can rapidly respond to changes in the glomerular environment [7]–[10]. Mutations in a number of podocyte proteins have been shown to cause rearrangement of the actin cytoskeleton and subsequent proteinuria [11]–[14]. Synatopodin, an actin-binding protein, is expressed at high levels in podocytes and plays a key role in stabilizing the actin cytoskeleton [7]. Indeed, loss of synaptopodin expression is a common feature in podocyte damage and glomerular injury [7], [10]. In addition, mice with mutations in synaptopodin are highly susceptible to podocyte damage and glomerular injury, as shown by the prolonged proteinuria seen when challenged with a dose of lipopolysaccharide that causes only transient proteinuria in wild type mice [10]. Synaptopodin modulates actin organization and cell motility through regulation of RhoA signalling [7]. Recently, Faul et al [8] demonstrated that phosphorylation of synaptopodin enables it to bind to 14-3-3β, which protects synaptopodin from cathepsin L-mediated degradation. In addition, it was shown that cyclosporine A can prevent de-phosphorylation of synaptopodin resulting in maintenance of the synaptopodin/14-3-3β interaction and normal synaptopodin function, thereby protecting mice from lipopolysaccharide-induced transient proteinuria [8].

14-3-3 is a family of dimeric proteins that can interact with a wide range of target proteins [15]. The association or disassociation of 14-3-3 with its target proteins participates in the regulation of many cellular processes, including apoptosis, cell division, transcription, trafficking and regulation of cytoskeletal protein [16], and may be involved in pathogenesis of different human diseases [17]. 14-3-3 proteins specifically bind to phosphoserine or phosphothreonine residues on target proteins to regulate cellular processes.

Previous studies have shown that RvD1 and/or RvE1 can suppress acute damage to the tubulointerstitial compartment of the kidney in models of renal ischemia/reperfusion injury and unilateral ureteric obstruction [18], [19]. However, it is not known whether RvD1 treatment can prevent or halt glomerular disease, and in particular whether RvD1 can protect podocytes from damage and loss of synaptopodin expression. To address this important question, examined a mouse model of adriamycin (ADR)-induced nephropathy in which podocyte damage is an early event leading to a rapid onset of proteinuria and development of lesions resembling human focal and segmental glomerulosclerosis [20].

## Materials and Methods

### Experimental Animals

At 8 weeks of age, BALB/c male mice (25 to 30 g body weight) received a single intravenous injection of ADR (10.5 mg/kg; Sigma, St. Louis, MO) [20]. Control animals were administered an equivalent intravenous volume of normal saline (NS) vehicle. ADR or NS mice were sacrificed at 1, 2 and 4 weeks after injection (*n*  = 6/group/time point). In the treatment study, mice received daily intraperitoneal injections of RvD1 (4 ng/g body weight/day) starting either 30 minutes (early treatment) or 14 days after ADR injection (late treatment), and continued until animals were killed on day 28 (*n*  = 6/group). The ADR control group received daily injections of vehicle (saline) for 28 days. The RvD1 dose regimen was based on our previous study [18]. In a second study, 17R-RvD1 (4 ng/g body weight/day) or vehicle was administered from day 14 after ADR injection until animals were killed on day 28. Urine, blood and kidney samples were collected from each animal. Each kidney was divided into three parts for (1) immunoprecipitation/Western blotting, (2) 10% buffered formalin fixed, paraffin-embedded tissue, (3) 4% paraformaldehyde-fixed, OCT-embedded tissue. All experiments were performed with the approval of the Animal Ethics Committee of Monash University, Australia.

### Measurement of Proteinuria and Creatinine

Measurement of urine protein and creatinine were determined using a detergent compatible protein assay kit (Bio-Rad, Hercules, CA) and creatinine assay kit (Cayman Chemical, Ann Arbor, MI) according to instructions. Proteinuria was normalized for creatinine excretion. Blood samples taken from mice at the time of sacrifice were used to determine serum creatinine levels using the creatinine assay kit.

### Plasmids

pcDNA-Flag-HA-14-3-3β and pCMV-SPORT6-synaptopodin were purchased from Addgene org (Cambridge, MA) and DNAFORM (Yokohama, Kanagawa, JAPAN), respectively. The lysine acetylation site point mutations K51/Q, K77/Q and K117+122/Q were obtained by site-directed mutagenesis using **Stratagene's QuikChange™ Site-Directed Mutagenesis Kit** (La Jolla, CA). Following primers were used for site-directed mutagenesis: 14-3-3-K51/Q-R: 5′-RGAGAGAAATCTGCTCTCTGTTGCCTACCAGAATGTGG-3′; 14-3-3-K51/Q-L: 5′-ATTCTGGTAGGCAACAGAGAGCAGATTTCTCTCTTCGTTG-3′; 14-3-3-K77/Q-R: 5′GGAATGAGAAGCAGCAGCAGATGGGCAAAGAGTACCGTG-3′; 14-3-3-K77/Q-L: 5′-CCATCTGCTGCTGCTTCTCATTCCTCTCTGTTTTCTG-3′; 1433K117-122/Q-R: 5′- AAAGTCAGGTGTTCTACTTGCAAATGAAAGGAGATTATTTTAGG-3′; 14-3-3K117-122/Q-L: 5′CCTTTCATTTGCAAGTAGAACACCTGACTTTCTGGTTGTGTAGC-3′. The point mutations were verified by sequencing.

### Antibodies

The following antibodies were used for IP, Western blot analysis and/or immunohistochemistry: rabbit anti-ALX (Santa Cruz Biotechnology, Dellas, Texas), rabbit anti-fibronectin (Sigma-Aldrich, Castle Hill, Australia); mouse anti-α-smooth muscle actin conjugated with Cy3 (α-SMA-Cy3; Sigma Chemical Co.), rabbit anti-α-tubulin conjugated with horseradish peroxidase (HRP) (Cell Signalling Technology, Beverly, MA), rabbit anti-acetylated lysine antibody (Cell Signaling Technology), goat anti-collagen type IV (Southern Biotechnology, Birmingham, AL), mouse anti-14-3-3β antibody (Abcam, Cambridge, UK), mouse anti-glyceraldehyde 3-phosphate dehydrogenase conjugated with HRP (GAPDH-HRP; Chemicon, Temecula, CA), peroxidase-conjugated rabbit anti-goat, rabbit anti-mouse IgM and goat anti-rabbit IgG (Sigma-Aldrich), HRP-conjugated mouse anti-rabbit IgG and rabbit anti-mouse IgG (Cell Signalling Technology). The PhosphoDetect™ Phosphoserine Detection Kit (Calbiochem, Billerica, MA) was used to detect phosphor-serine in synaptopodin.

### Immunoprecipitation (IP)/Western Blot Analysis

At 1, 2 and 4 weeks after NS or ADR injection, or 4 weeks after ADR injection with/without treatment, the kidneys were homogenized and suspended in 0.4 ml of lysis buffer containing 10 mmol/L Tris-HCL, pH 7.4, 1% Triton X-100, 0.5% deoxycholate, 1 mmol/L phenylmethyl sulfonyl fluoride, and 10% proteinase inhibitor (Roche, Castle Hill, Australia). Protein concentration estimations were performed with a detergent-compatible protein assay kit (Bio-Rad). 500 µg of total protein was used to perform the IP. The following antibodies were used to perform IP: rabbit anti- hemagglutinin (HA) tag antibody (Cell Signalling Tachnology, 1∶100), mouse anti-14-3-3β antibody (Abcam, 1∶100) and rabbit anti-synaptopodin (Bioss Inc, Woburn, MA; 1∶100). Fifty µg of total protein was loaded per well and separated by sodium dodecyl sulfate-polyacrylamide gel electrophoresis on a 10% polyacrylamide gel. Gels were electroblotted onto a polyvinylidene difluoride membrane (Roche). Blots were incubated with either anti-14-3-3β (1∶1000), anti-p-serine (1∶10000), anti-acetylated lysine (1∶1000), anti-HA (1∶10000), anti-ALX (1∶200) or anti-synaptopodin (1∶10000) in 5% bovine serum albumin (BSA) in wash buffer overnight at 4°C. Blots were then incubated with peroxidase-conjugated mouse anti-rabbit (1∶160,000), rabbit anti-mouse (1∶160,000), or goat anti- rabbit IgG (1∶160,000) for 1 hour at room temperature, and bound antibody was detected by ECL Plus (Amersham, Little Chalfont, UK) and captured on autoradiography film (Amersham). To confirm protein levels loaded, membranes were reprobed by anti-α-tubulin-HRP (1∶20,000) or anti-GAPDH-HRP (1∶80,000). Densitometry analysis was performed by a Gel Pro analyzer program (Media Cybernetics, Silver Spring, MD).

### Histology and Confocal Microscopy

Renal histology was examined in 10% buffered formalin fixed, paraffin-embedded tissue sections (4 µm) stained with periodic acid-Schiff (PAS) and Masson’s trichrome. The degree of glomerulosclerosis was defined by the presence of PAS-positive staining material involving >30% of each glomerulus. Eighty to one-hundred glomeruli per animal from random, non-overlapping cortical regions were scored to determine the percentage of glomeruli displaying glomerulosclerosis [21]. Interstitial fibrosis was quantified by point counting of the trichrome-stained interstitium and expressed as a percentage of the total cortical area [22]. The mean value of five cortical fields was determined for each section. Five sections were selected from each kidney. All scoring was performed on blinded slides.

Cryostat sections of tissues fixed in 4% paraformaldehyde (Sigma-Aldrich) were blocked with 2% BSA in PBS and incubated with the following antibodies: Cy3-conjugated mouse anti-α-SMA antibody, rabbit anti-fibronectin (Sigma-Aldrich) followed by goat anti-rabbit Alexa Fluor 488; rabbit anti-synaptopodin followed by goat anti-rabbit Alexa Fluor 555; goat anti-collagen IV (Southern Biotechnology, Birmingham, AL, USA), followed by chicken anti-goat AlexaFluor 647 (Invitrogen) and rat anti-mouse F4/80 antibody (Serotec, Oxford, UK) followed by goat anti-rat AlexaFluor 488 antibody (Invitrogen). Sections were counterstained with 4,6-diamidino-2-phenylindole (Sigma-Aldrich) to visualize nuclei. Sections were analysed with an OlympusFluoview 1000 confocal microscope (Olympus, Tokyo, Japan), FV10-ASW software (v 1.3c; Olympus) and an oil UPLFL ×60 objective (NA 1.25; Olympus). The number of infiltrating interstitial macrophages was quantified in 20 non-overlapping cortical fields and expressed as cells per mm^2^ of cortical interstitium. The area of interstitial staining for α-SMA, collagen IV and fibronectin were assessed by image analysis (excluding large vessels). All scoring was performed on blinded slides.

### Mouse Podocyte Culture

Podocytes between passage 10 and 15 were maintained in RPMI 1640 medium supplement with 10% fetal bovine serum (FBS) and 1% streptomycin/penicillin solution [23]. Cells were propagated in 10 U/ml murine IFNγ at 33°C and then differentiated by culture for 7 days at 37°C in the absence of IFNγ [23]. Differentiated podocytes showed prominent cytoplasmic processes and expressed synaptopodin. Differentiated podocytes were stimulated with TNF-α 10 ng/ml, RvD1 4 ng/ml or TNF-α 10 ng/ml+RvD1 4 ng/ml for 6 and 12 hrs before harvesting. In a separate study, differentiated podocytes were stimulated with TNF-α 10 ng/ml, RvD1 4 ng/ml, WRW^4^ 10 µM, TNF-α 10 ng/ml+RvD1 4 ng/ml, TNF-α 10 ng/ml+WRW^4^ 10 µM or TNF-α 10 ng/ml+RvD1 4 ng/ml+WRW^4^ 10 µM for 24 h before harvesting.

### Co-transfection and Immunoprecipitation

HEK293T cells (ATCC, Manassas, Va) were transfected in triplicate with pcDNA-Flag-HA-14-3-3β (Addgene org, Cambridge, MA) and pCMV-SPORT6-synaptopodin (DNAFORM, Yokohama, Kanagawa, JAPAN) using lipofectamine 2000 (Invitrogen, Vic, Australia); 48 h after transfection, the transfected cells were subjected to stimulation with TNF-α 10 ng/ml for 1 h, with or without 4 ng/ml RvD1. Cells were harvested for immunoprecipitation and immunoblotting.

HEK293T cells were transfected in triplicate with pcDNA-Flag-HA-14-3-3β, pcDNA-Flag-HA-14-3-3β with lysines K51, 77, 117 and 122 replaced with Q and pCMV-SPORT6-synaptopodin using lipofectamine 2000 (Invitrogen); 48 h after transfection, cells were harvested for immunoprecipitation and immunoblotting.

### Real-time PCR

Total RNA from cultured cells and kidney samples was isolated using High Pure RNA Isolation Kits (Roche Applied Science, Castle Hill, NSW, Australia). RNA reverse transcription used the SuperScript® III First-Strand Synthesis System (Invitrogen), and real-time PCR was performed using SYBR Green PCR Reagents (Sigma-Aldrich, Castle Hill, NSW, Australia). Primers were as follows: mouse lipoxin A4 receptor (ALX), 5′-CCATGCTCACTGTCAGAGGA-3′ and 5′- GCTGTTGAAGAAAGCCAAGG-3′; mouse arginase I, 5′-CGCCTTTCTCAAAAGGACAG-3′ and 5′- CCAGCTCTTCATTGGCTTTC-3′; mouse YM1, 5′- TGGAGGATGGAAGTTTGGAC-3′and 5′- AATGATTCCTGCTCCTGTGG-3′; mouse monocyte chemotactic protein-1 (MCP-1), 5′-CAAGAAGGAATGGGTCCAGA-3′ and 5′-AAGGCATCACAGTCCGAGTC-3′; mouse TNFα, 5′-AGCCCCCAGTCTGTATCCTT-3′ and 5′-CTCCCTTTGCAGAACTCAGG-3′; mouse synaptopodin, 5′- TAACTTCCGTGGAGCTGCTT -3′ and 5′- GACAGATGTTGTAGCCGAGGA -3′, and mouse glyceraldehyde-3-phosphate dehydrogenase (*GAPDH*), 5′-CAGATCCACAACGGATATATTGGG-3′ and 5′-CATGACAACTTTGGCATTGTGG-3′. Reaction specificity was confirmed by electrophoresis analysis of products with bands of the expected size detected. The relative amount of mRNA was calculated using the comparative *C*T (Δ*C*T) method and expressed as mean ± SD.

### Statistical Analysis

Data are presented as mean ±1 SD with statistical analyses performed using one-way analysis of variance with Tukey’s post-test analysis (GraphPad Prism 3.0, GraphPad Software, Inc., San Diego, CA).

## Results

### RvD1 Administration Reduced Proteinuria and Improved Renal Function in ADR-induced Nephrosis

**Figure 1:**
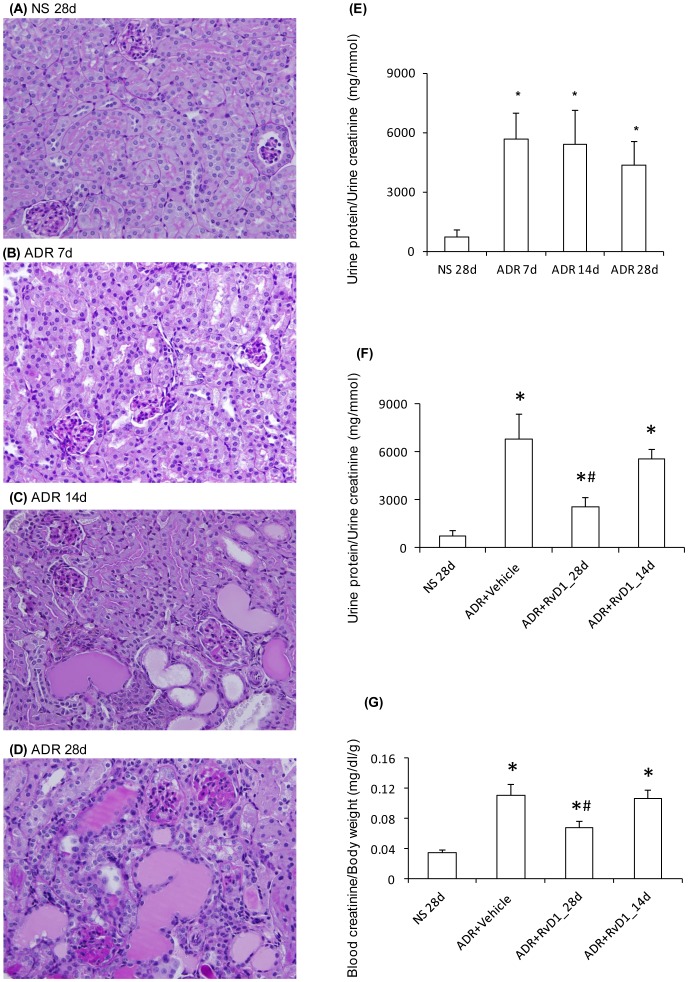
Characterization of ADR-induced nephropathy. PAS staining of sections from mouse kidneys: (A) 28 days after normal saline (NS) injection, or (B) 7 days, (C), 14 days, and (D) 28 days, after ADR injection. (E) Urine protein to creatinine ratio over the time-course of ADR pathropathy. (F) Urine protein to creatinine ratio on day 28 of ADR nephropathy after early (28d) or late (14d) RvD1 treatment. (G) Renal function assessed by blood creatinine to body weight on day 28 of ADR nephropathy after early (28d) or late (14d) RvD1 treatment. Data are mean ± SD, n = 6. ^*^ *P*<0.05 *vs* NS 28d; ^#^ *P*<0.05 *vs* ADR+vehicle and *vs* ADR+RvD1_14d. Original magnification, x400 (A–D).

Administration of ADR to mice resulted in heavy proteinuria by day 7 which continued to day 28 when mice were killed (Fig. 1E). On day 7, mouse kidneys had a relatively normal appearance by PAS staining; however, by day 14 some glomeruli showed focal areas of glomerulosclerosis plus tubular dilation and cast formation were evident. The degree of glomerulosclerosis was substantially worse by day 28, with worsening tubular damage and interstitial mononuclear cell infiltration (Fig. 1A–D). A significant loss of renal function was also evident on day 28 as shown by increased blood creatinine levels (Fig. 1G).

### RvD1 Ameliorated the Development of Renal Fibrosis in ADR-induced Nephropathy

**Figure 2:**
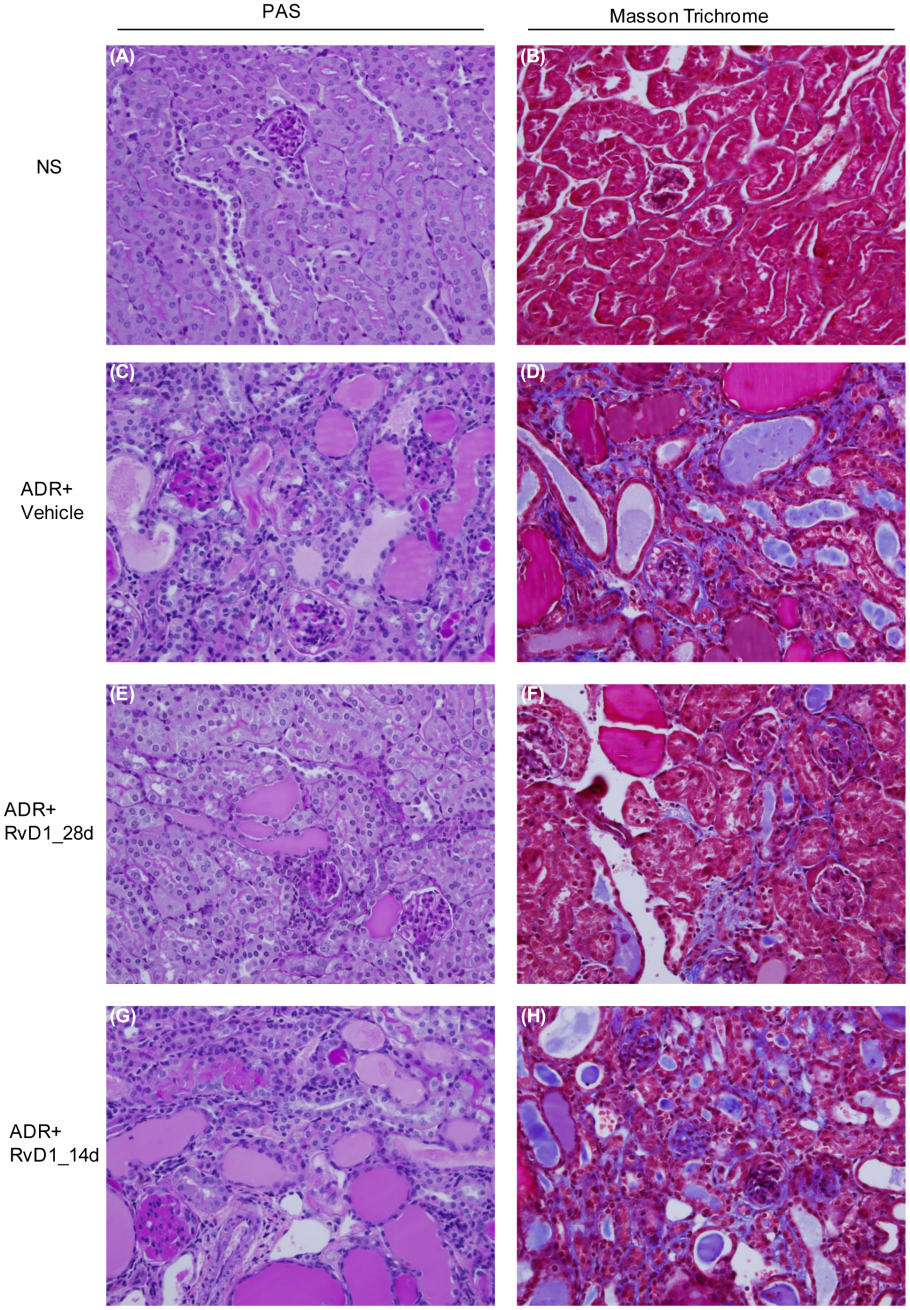
Effect of RvD1 treatment on renal pathology in ADR nephropathy. PAS and Masson trichrome staining of mouse kidney sections: (A, B) normal saline (NS) control; (C,D) day 28 ADR with Vehicle treatment; (E,F) day 28 ADR with early RvD1 treatment, and; (G,H) day 28 ADR with late RvD1 treatment. Early but not late administration of RvD1 ameliorated development of glomerulosclerosis and interstitial fibrosis. Original magnification, x400.

**Figure 3:**
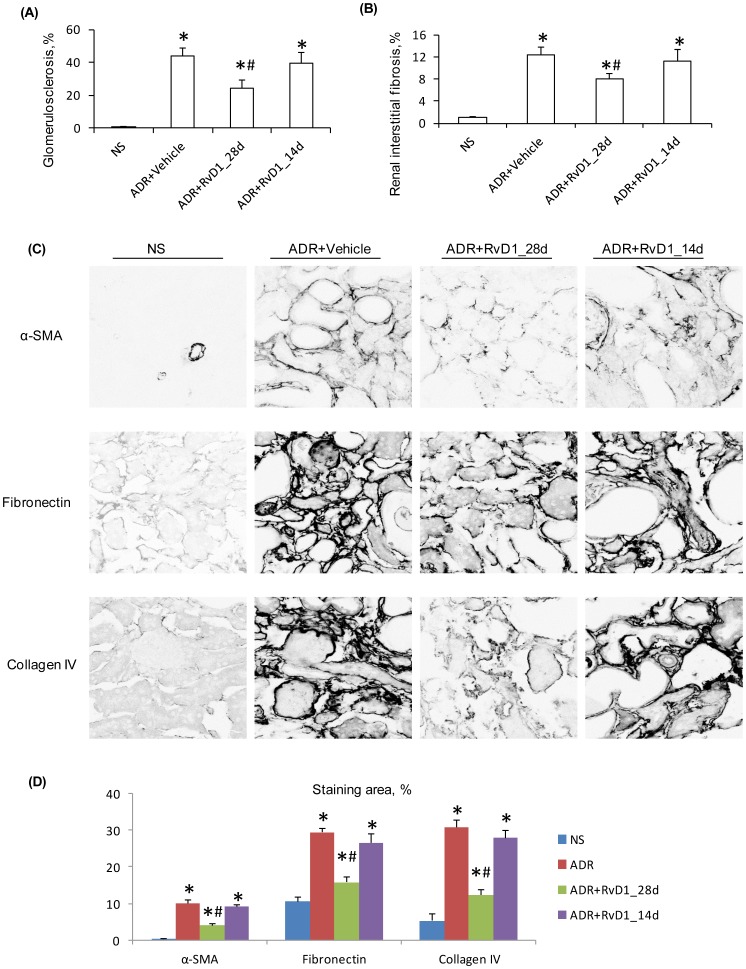
Effect of RvD1 treatment on kidney fibrosis in ADR nephropathy. Graphs of (A) glomerulosclerosis, and (B) interstitial fibrosis, for normal saline (NS) controls, and day 28 ADR with vehicle treatment or with early (28d) or late (14d) RvD1 treatment. (C) Confocal microscopy showing immunostaining for α-smooth muscle actin (α-SMA), fibronectin and collagen IV expression in the above experimental groups in ADR nephropathy. (D) Graph quantifying the area of interstitial staining for α-SMA, fibronectin and collagen IV in ADR nephropathy. Data are mean ± SD, n = 6. ^*^ *P*<0.05 versus NS; ^#^ *P*<0.05 *vs* ADR+Vehicle and *vs* ADR+RvD1_14d. Original magnification, x600 (C).

Early RvD1 treatment substantially inhibited the development of proteinuria and significantly improved renal function by day 28 in ADR nephropathy (Fig. 1F&G). However, late RvD1 treatment over days 14 to 28 failed to modify ongoing heavy proteinuria and did not affect renal dysfunction (Fig. 1F&G). Early RvD1 treatment significantly reduced the development of glomerulosclerosis, tubular damage, cast formation and interstitial fibrosis (Fig. 2 and Fig. 3A&B). However, late RvD1 treatment failed to halt progressive glomerulosclerosis, tubular damage and interstitial fibrosis (Fig. 2G&H, Fig. 3A&B). Immunostaining identified a significant increase in interstitial α-SMA+ myofibroblasts and interstitial deposition of collagen IV and fibronectin in ADR nephropathy (Fig. 3C&D). Early RvD1 treatment substantially reduced α-SMA+ myofibroblast accumulation and matrix deposition; however, late RvD1 treatment did not affect interstitial fibrosis (Fig. 3C&D).

**Figure 4:**
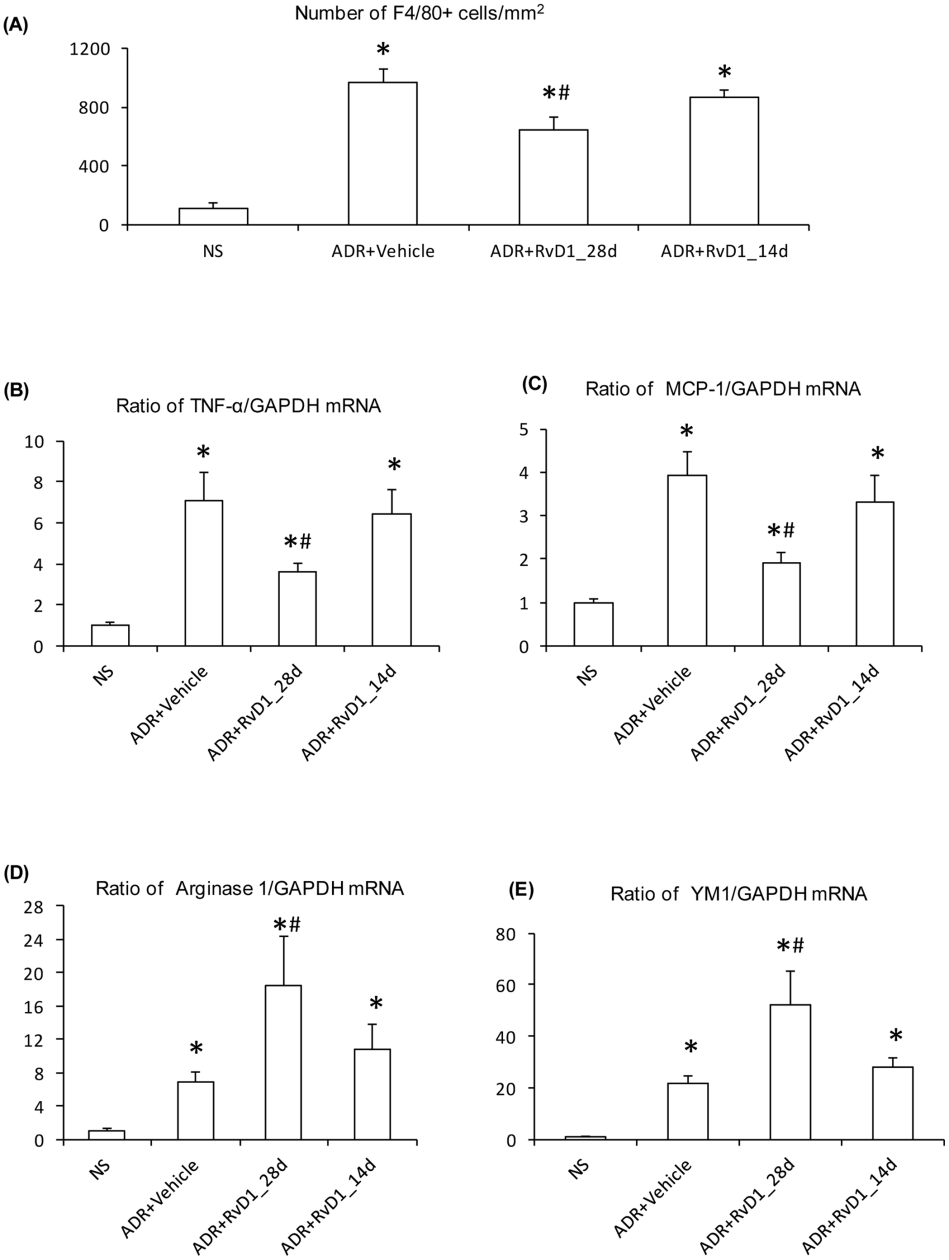
Effects of RvD1 treatment on macrophage infiltration in ADR nephropathy. (A) Quantification of F4/80+ macrophages/mm^2^ for normal saline (NS) controls, and day 28 ADR with vehicle treatment or with early (28d) or late (14d) RvD1 treatment. Real-time RT-PCR analysis of kidney mRNA levels of: (B) TNF-α; (C) MCP-1; (D) Arginase 1, and; (E) YM1. Data are mean ± SD, n = 6. *P<0.05 *vs* NS; #P<0.05 *vs* ADR+Vehicle or ADR+RvD1_14d.

A marked infiltrate of F4/80+ macrophages was evident on day 28 of ADR nephropathy. This was partially reduced by early RvD1 treatment but late RvD1 treatment had no effect upon macrophage numbers (Fig. 4A). Real-time PCR analysis of whole kidney tissue identified up-regulation of mRNA levels of the pro-inflammatory molecules TNF-α and MCP-1 (also considered markers of an M1-type phenotype) which was reduced by early but not late RvD1 treatment (Fig. 4B&C). In contrast, mRNA levels of arginase-1 and YM1 (considered markers of M2-type macrophages) were further up-regulated by early but not late RvD1 treatment (Fig. 4 D&E).

### RvD1 Prevents Loss of Synaptopodin Expression in ADR Nephropathy

**Figure 5:**
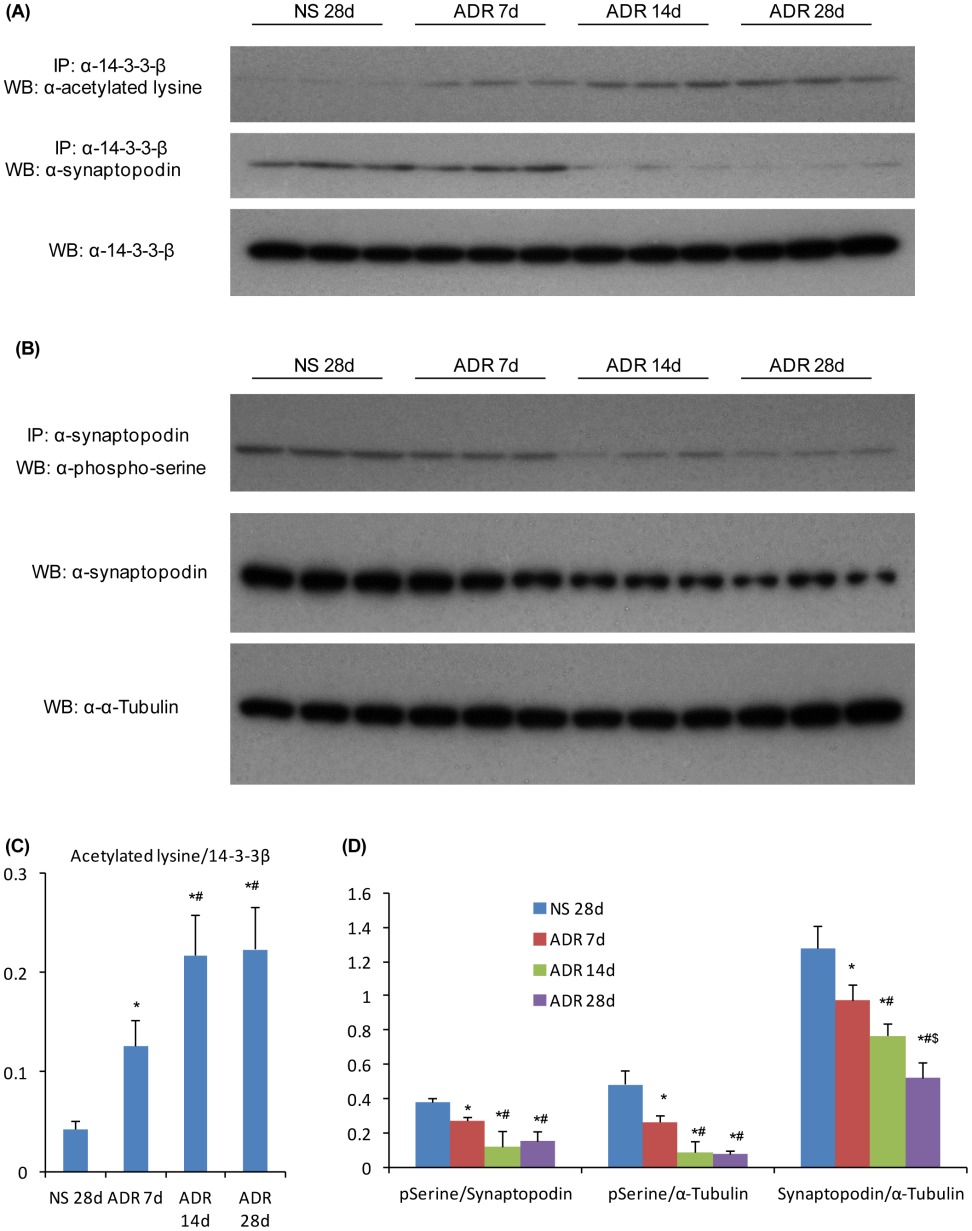
Induction of lysine acetylation in 14-3-3β and reduction of serine phosphorylation in synaptopodin in ADR nephropathy. Whole kidney lysates from normal saline treated mice or from mice 7, 14 or 28 days after ADR administration were examined by immunoprecipitation (IP)/Western blotting (WB) analysis. (A) IP for 14-3-3β followed by (top blot) WB for acetylated lysine showing a progressive increase in 14-3-3β acetylation in ADR nephropathy, or (middle blot) WB for synaptopodin showing binding between 14-3-3β and synaptopodin, and (bottom blot) WB for 14-3-3β showing equivalent IP of 14-3-3β from all lysates. (B) IP for synaptopodin followed by (top blot) WB for phosphor-serine showing a progressive loss of synaptopodin phosphorylation in ADR nephropathy, or (middle blot) WB for synaptopodin showing a progressive loss of synaptopodin expression in the progression of ADR nephropathy; (bottom blot) lysates were probed for α-tubulin to confirm equivalent protein content. (C) Graph shows densitometric analysis of the ratio of acetylated lysine to total 14-3-3β. Data are mean ± SD, n = 6. ^*^P<0.05 vs NS 28d; *^#^P<0.05* vs ADR 7d. (D) Graphs show densitometric analysis of the ratio of phospho-serine to total synaptopodin, the ratio of phospho-serine to α-tubulin, and the ratio of synaptopodin to α-tubulin (D). Data are mean ± SD, n = 6. ^*^P<0.05 vs NS 28d; *^#^P<0.05 vs* ADR 7d; ^$^ *P<0.05 vs* ADR 14d.

**Figure 6:**
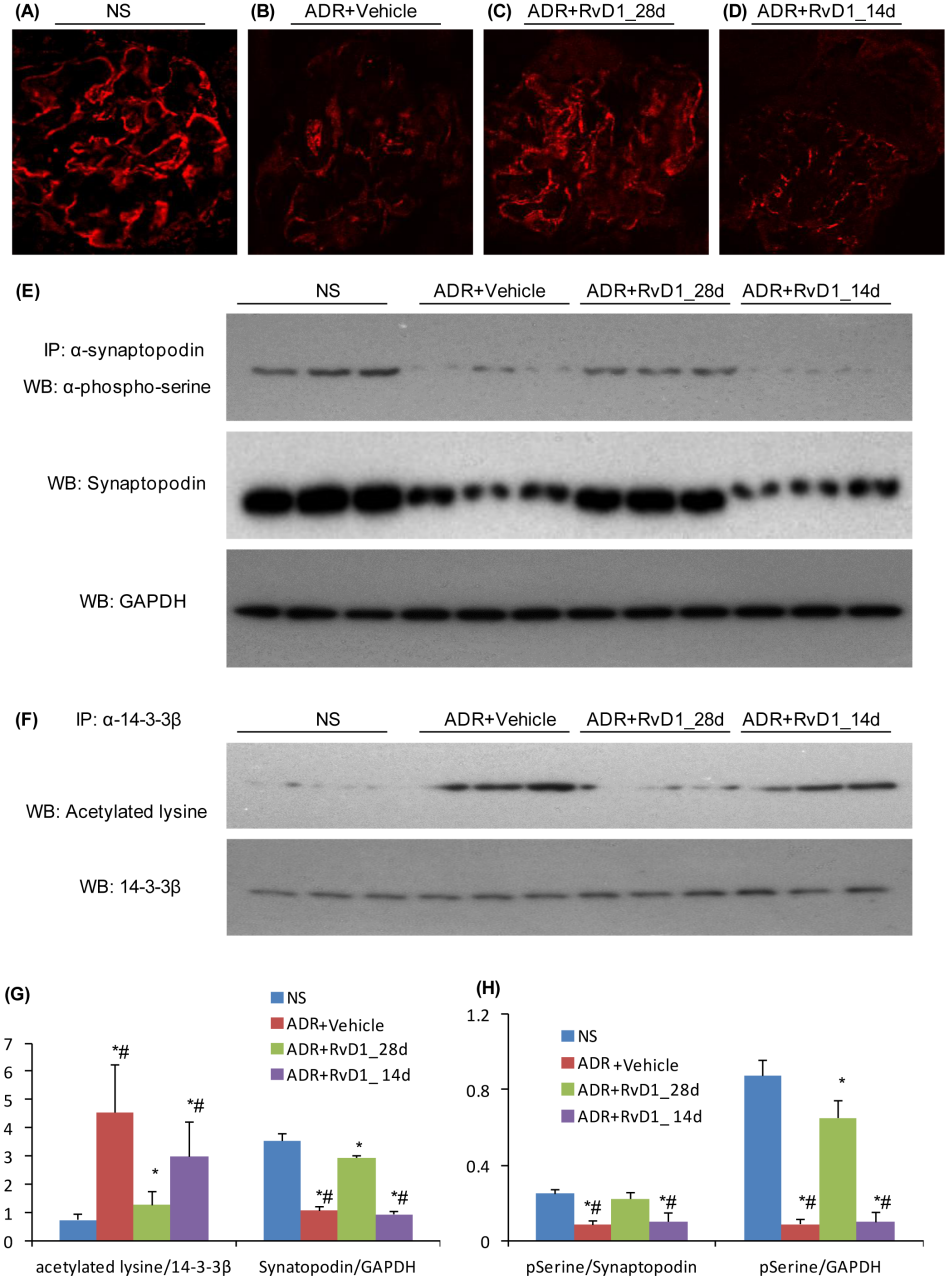
The effects of RvD1 treatment on 14-3-3β acetylation, synaptopodin phosphorylation and synaptopodin expression in ADR-induced nephrosis. Immunofluorescence staining of synaptopodin in mouse kidney sections: (A) normal saline (NS) control; (B) day 28 ADR with Vehicle treatment; (C) day 28 ADR with early RvD1 (28d) treatment, and; (D) day 28 ADR with late (14d) RvD1 treatment. (E) Kidney tissue lysates were immunoprecipitated (IP) for synaptopodin followed by (top blot) WB for phospho-serine, or (middle blot) WB for total synaptopodin, and (bottom blot) lysates were probed for GAPDH to confirm equivalent protein content. (F) Immunoprecipitation of kidney tissue lysates for 14-3-3β followed by (top blot) WB for acetylated lysine, or (bottom blot) WB for total 14-3-3β to confirm synaptopodin, and (bottom blot) WB for 14-3-3β showing equivalent IP of 14-3-3β from all lysates. (G) Graphs show densitometric analysis of the ratio of acetylated lysine to total 14-3-3β and the ratio synaptopodin to GAPDH. (H) Graphs show densitometric analysis of the ratio of phospho-serine to total synaptopodin and the ratio of phospho-serine to α-tubulin. Data are mean ± SD, n = 6. ^*^P<0.05 vs NS; *^#^P<0.05* vs ADR+RvD1_28d. Original magnification, X600 (A–D).

The administration of ADR to mice caused a progressive loss of glomerular synaptopodin expression as shown by Western blotting and confocal microscopy (Figs 5B&D and Fig. 6A&B). This was accompanied by a significant increase in acetylation of lysine residues in 14-3-3β (Fig. 5A&C) and a reduction in serine phosphorylation in synaptopodin (Fig. 5B&D) and a loss of interaction between 14-3-3β and synaptopodin (Fig. 5A&D). Early RvD1 treatment prevented these changes in 14-3-3β acetylation and synaptopodin phosphorylation and maintained the 14-3-3β/synaptopodin interaction (Fig. 6). However, late RvD1 treatment was not able to reverse the ADR-induced loss in synaptopodin expression or changes in 14-3-3β and synaptopodin post-translational modifications (Fig. 6).

**Figure 7:**
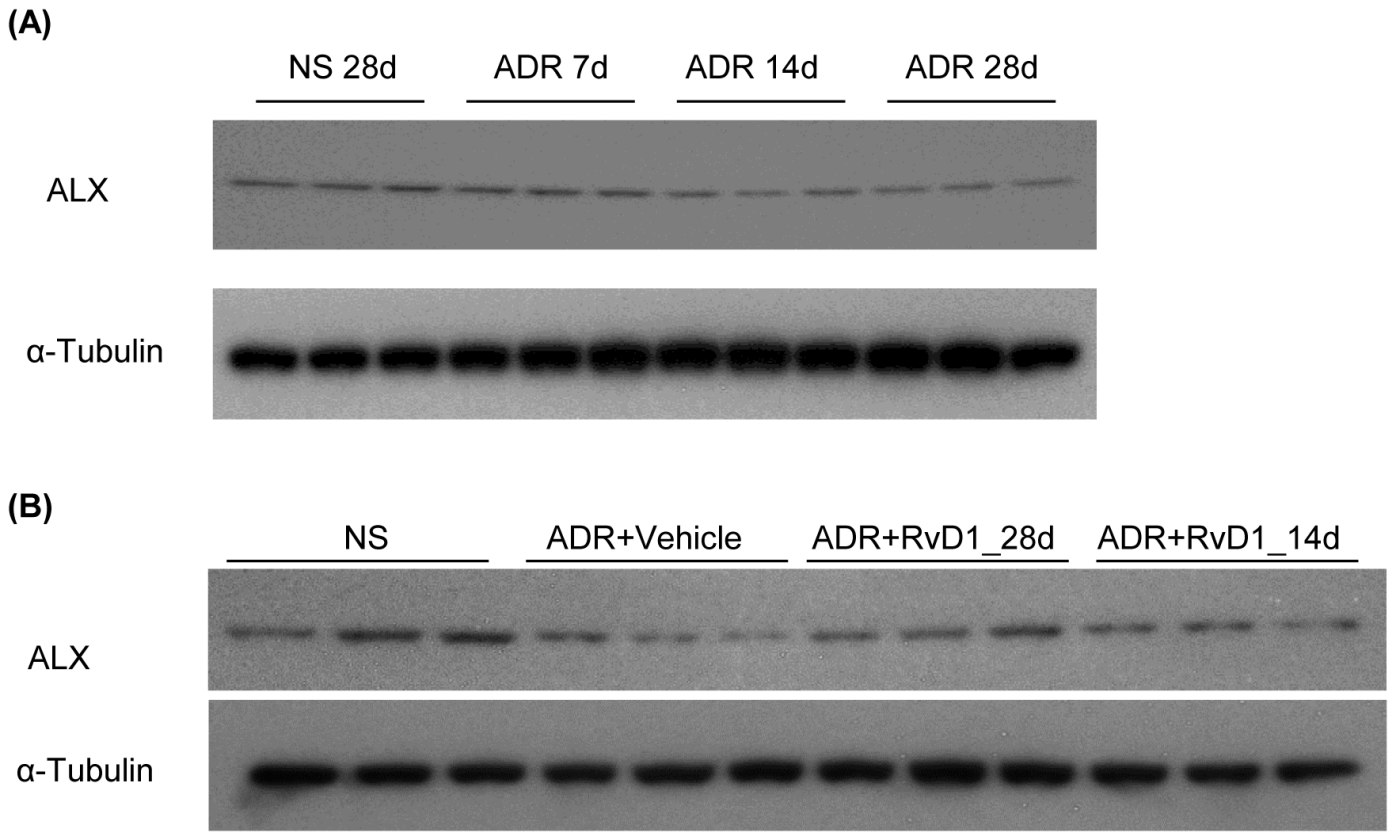
The protective effects of RvD1 on podocytes through ALX. Western blotting demonstrated the expression levels of ALX in whole kidney lysates from normal saline treated mice or from mice 7, 14 or 28 days after ADR administration (A) or from vehicle or RvD1 early or late treatment after ADR administration (B).

To investigate this further, we examined expression levels of ALX, one of the known RvD1 receptors, in ADR nephropathy. A time-course study showed a reduction in ALX levels on days 14 and 28 of ADR nephropathy (Fig. 7A). Early treatment with RvD1 from the start of ADR nephropathy prevented the reduction in ALX levels, but late treatment was unable to prevent this (Fig. 7B).

**Figure 8:**
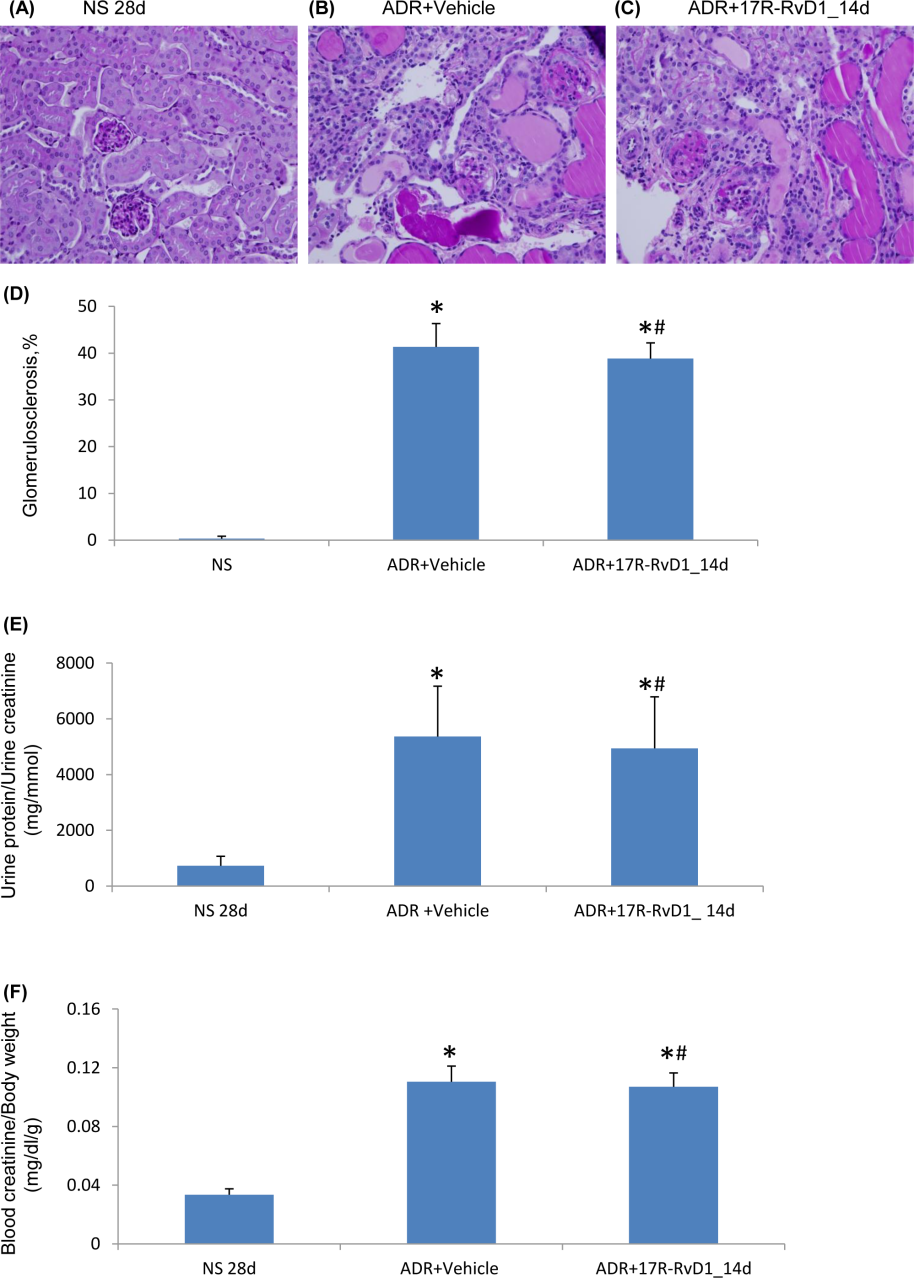
Effect of 17R-RvD1 treatment on ADR nephropathy. PAS staining of mouse kidney sections: (A) normal saline (NS) control; (B) day 28 ADR with Vehicle treatment and (C) day 28 ADR with late 17R-RvD1 treatment. Original magnification, x400. Graphs of (D) glomerulosclerosis, (E) proteinuria and (F) serum creatinine. Data are mean ± SD, n = 6. ^*^ *P*<0.05 versus NS; ^#^ *P*>0.05 *vs* ADR+Vehicle.

One possible reason for the failure of late RvD1 treatment in ADR nephropathy is that this compound can be rapidly inactivated by eicosanoid oxidoreductases [2]. Therefore, we tested whether 17(R)-RvD1, which is resistant to such inactivation [2], could provide protection in established ADR nephropathy. However, late administration of 17(R)-RvD1 over days 14 to 28 administration also failed to give protection in ADR nephropathy in terms of renal fibrosis (Fig. 8A–D), proteinuria (Fig. 8E) and loss of renal function (Fig. 8F). The activity of 17(R)-RvD1 was confirmed in studies of cultured podocytes (data not shown).

### RvD1 Prevents TNF-α-induced Loss of Synaptopodin in Cultured Podocytes

**Figure 9:**
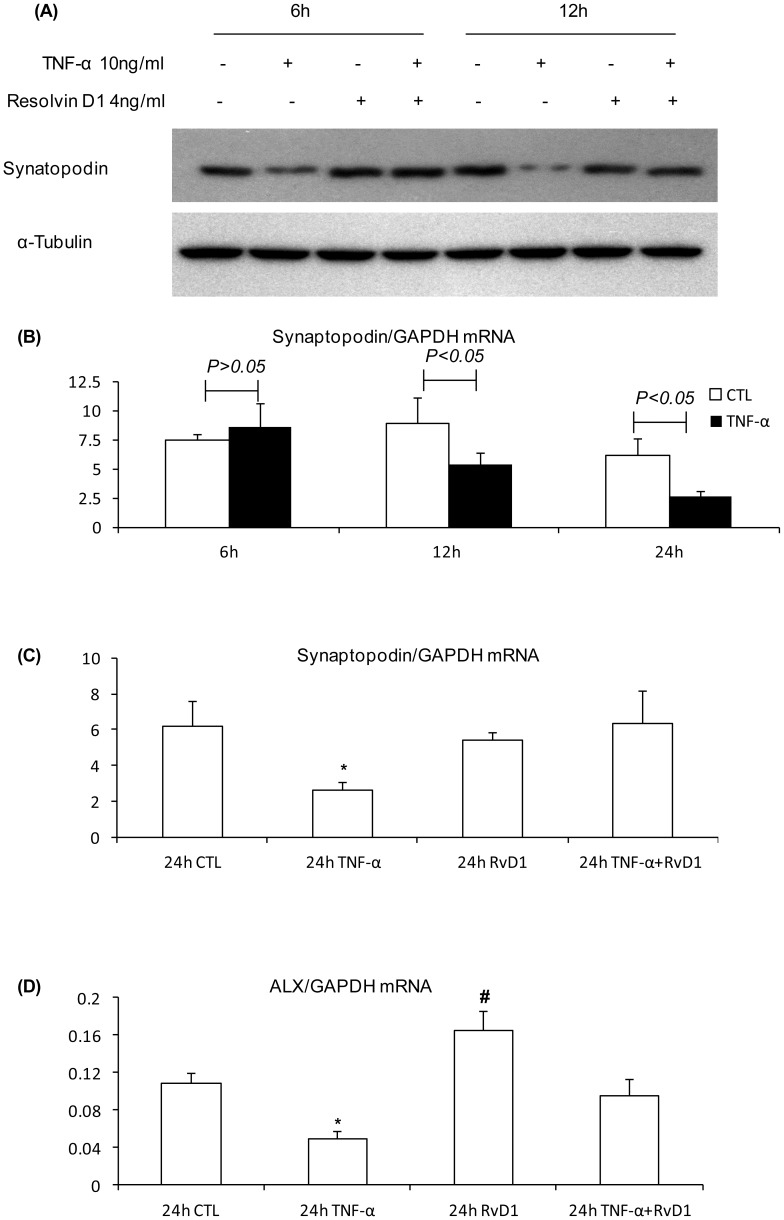
RvD1 protected TNF-α-induced loss of synaptopodin in mouse podocytes. (A) Podocytes were cultured for 6 or 12 hr with TNF-α, RvD1 or both together. The Western blot shows protein levels for synaptopodin and the α-tubulin control. (B) Real-time RT-PCR analysis of synaptopodin mRNA levels shows no change in mRNA levels 6 hr after TNF-α treatment, and a reduction in mRNA levels after 12 and 24 hr. (C) Real-time RT-PCR analysis shows RvD1 (4 ng/ml) can prevent the down-regulation of synaptopodin mRNA seen with 24 hr treatment with TNF-α (10 ng/ml). (D) Real-time RT-PCR analysis shows podocytes express ALX mRNA. This is down-regulated 24 hr after TNF-α treatment, which is prevented by RvD1, and RvD1 by itself up-regulates RLX mRNA levels. ^*^P<0.05 vs 24 h control (CTL), or 24 h RvD1, or 24 hTNF-α+RvD1; ^#^ *P*<0.05 *vs* 24 h CTL and *vs* 24 h TNF-α+RvD1.

**Figure 10:**
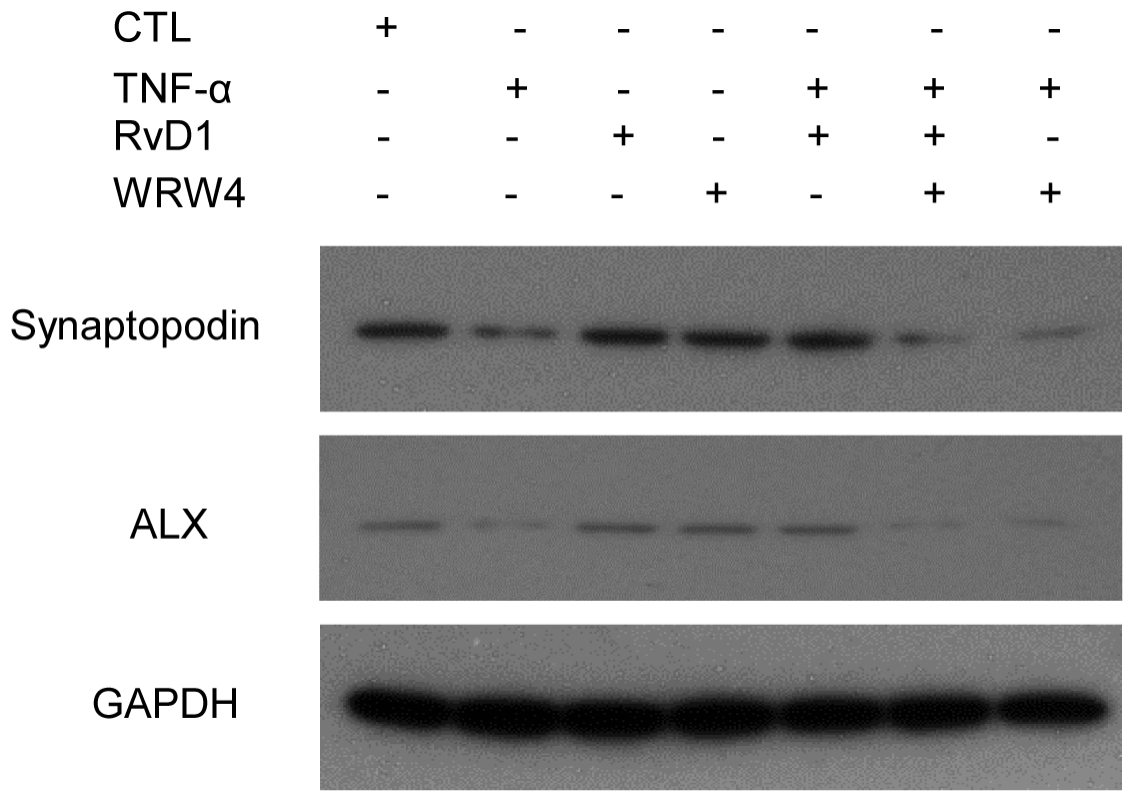
The effects of RvD1 through ALX. Western blotting demonstrating the expression levels of ALX after TNF-α, RvD1, WRW^4^ and different combination treatments in podocytes.

To determine whether RvD1 can directly affect synaptopodin expression we investigated a mouse podocyte cell line. Preliminary studies found podocytes to be extremely sensitive to the toxic effects of Adriamycin. Therefore, we studied the pro-inflammatory cytokine TNF-α, a well characterised podocyte toxin [24] which is also up-regulated in ADR nephropathy. Synaptopodin protein levels were down-regulated within 6 hr of TNF-α addition to cultured podocytes (Fig. 9A). Interestingly, this reduction in synaptopodin protein levels preceded the reduction in mRNA levels suggesting a post-translational mechanism (Fig. 9B). The addition of RvD1 prevented the TNF-α induced-loss of synaptopodin protein levels and down-regulation of synaptopodin mRNA (Fig. 9A&C). Real-time PCR showed mRNA expression of the RvD1 receptor ALX on podocytes (Fig. 9D). Further analysis demonstrated that TNF-α reduced ALX mRNA levels which was prevented by RvD1 (Fig. 9D). In addition, the ALX antagonist WRW^4^ abolished the protective effects of RvD1 on TNF-α-induced loss of synaptopodin in podocytes (Fig. 10), suggesting that the protective effects of RvD1 are ALX-dependent.

### RvD1 Prevents TNF-α-induced 14-3-3β and Synaptopodin Post-translational Modification

**Figure 11:**
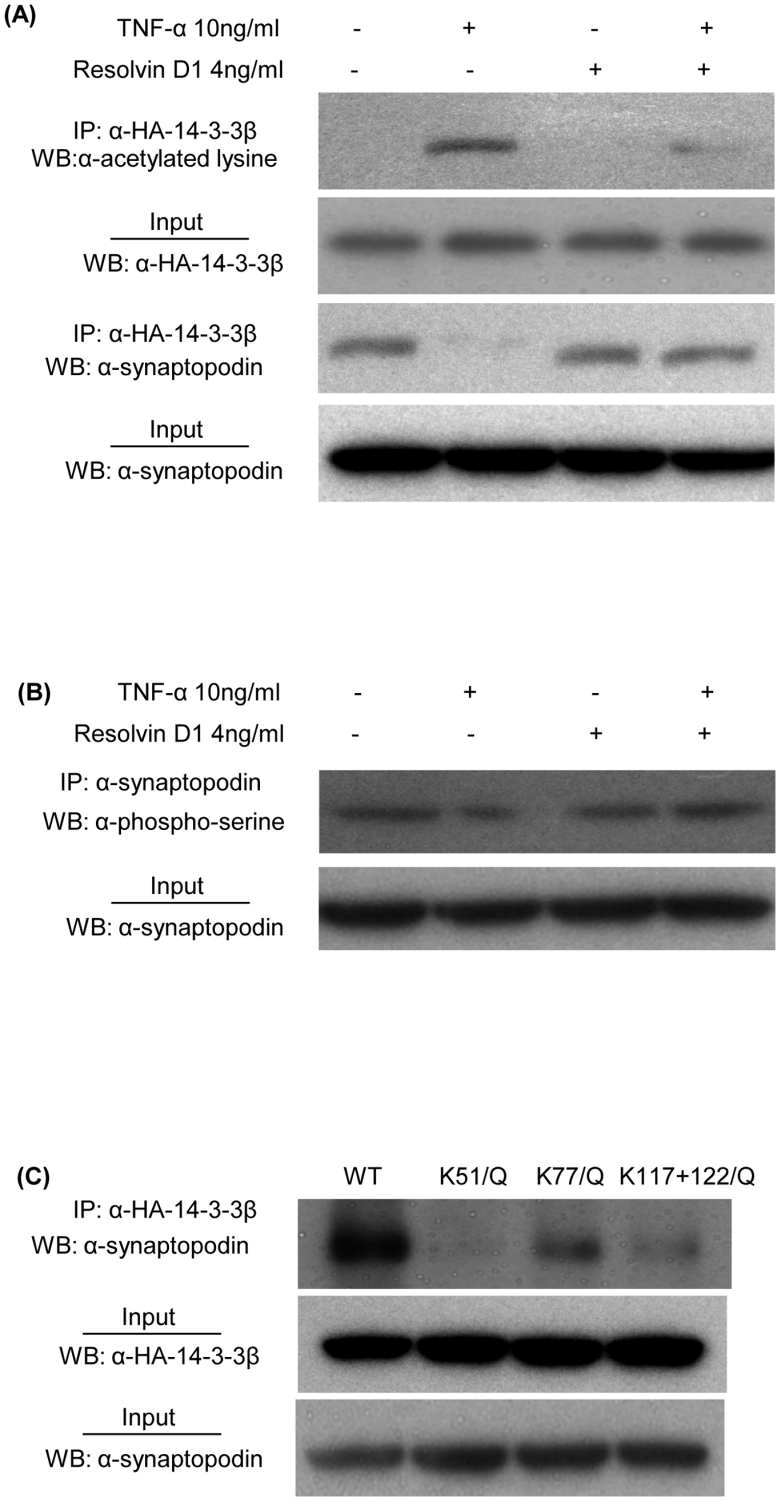
Acetylation of 14-3-3β interrupts binding to synaptopodin. Wild type HA tagged 14-3-3β and synaptopodin expression plasmids were co-transfected into HEK 293T cells, and 48 hr later cells were stimulated with TNF-α (10 ng/ml) for 60 minutes. (A) Immunoprecipitation (IP) of 14-3-3β followed by Western blotting (WB) for acetylated lysine shows TNF-α induced 14-3-3β acetylation which is prevented by RvD1 (top blot), with WB showing equivalent IP of 14-3-3β from all lysates (second blot). WB for synaptopodin shows a loss of binding to 14-3-3β in TNF-α stimulated cells which is prevented by RvD1 (third blot), and WB shows equivalent levels of synaptopodin in all lysates (bottom blot). (B) Immunoprecipitation of synaptopodin followed by WB for phospho-serine (top blot), and WB showing equivalent IP synaptopodin from all lysates (bottom blot). (C) HEL293T cells were separately co-transfected with the synaptopodin expression plasmid plus one HA-tagged 14-3-3β plasmid expressing either wild type 14-3-3β, or 14-3-3β in which K51, K77 or K117+K122 residues were replaced with glutamine (Q). Cell lysates made 48 hr later were immunoprecipitated for 14-3-3β followed by WB for synaptopodin (top blot) showing that 14-3-3β acetylation inhibited binding to synaptopodin, and WB of cells lysates showed equivalent expression of 14-3-3β (middle blot) and synaptopodin (bottom blot).

To examine the interaction between 14-3-3β and synaptopodin, we used plasmid-based transfection in HEK293T cells. Expression of HA-tagged 14-3-3β and synaptopodin was readily detected in HEK293T cells, and experiments showed that 14-3-3β was not acetylated and that 14-3-3β bound to synaptopodin (Fig. 11A). Unstimulated cells also showed serine phosphorylation of synaptopodin (Fig. 11B). TNF-α stimulation rapidly (within 1 hr) induced acetylation of 14-3-3β, suppressed synaptopodin phosphorylation, and caused dissociation of 14-3-3β/synaptopodin binding (Fig. 11A&B). These TNF-α-induced changes were prevented by RvD1, while RvD1 by itself had no effect upon the 14-3-3β/synaptopodin interaction (Fig. 11A&B).

### 14-3-3β Acetylation Inhibits Interaction of 14-3-3β with Synatopodin

To mimic acetylation of 14-3-3β, lysines at positions 51, 77, 117 and 122 were replaced with glutamine (Q) in HA-tagged 14-3-3β expression plasmids. Immunoprecipitation/Western blotting analysis showed that wild-type 14-3-3β strongly bound to synaptopodin; however, K51/Q and combined K117/Q plus K122/Q mutations virtually abolished 14-3-3β binding to synaptopodin, while the K77/Q mutation partially reduced 14-3-3β/synaptopodin binding (Fig. 11C). This demonstrates that acetylation of individual lysine residues in 14-3-3β is sufficient to block its interaction with synaptopodin.

## Discussion

This is the first study to demonstrate that RvD1 can suppress glomerular injury. This is a significant advance on previous studies showing that RvD1 and/or RvE1 can suppress acute tubulointerstitial injury on the basis that glomerular injury, and podocyte damage in particular, are primary pathologic events in the development of most forms of progressive kidney disease.

The ability of RvD1 to suppress development of ADR nephropathy can be attributed in large part to protecting against podocyte damage. Podocyte damage is an early event following ADR administration and the early onset of heavy proteinuria coincided with a significant loss of synaptopodin protein expression even though glomeruli had a relatively normal appearance by light microscopy. Therefore, it is likely that early RvD1 treatment was protective by preventing progressive podocyte damage following the ADR insult. This conclusion is supported by several pieces of data: (i) the down-regulation of synaptopodin expression evident on day 7 and 14 of ADR nephropathy was prevented by RvD1 treatment; (ii) the development of glomerulosclerosis, thought to be secondary to podocyte damage, was prevented by RvD1 treatment, and; (iii) RvD1 had a direct protective effect in preventing TNF-α-induced down-regulation of synaptopodin in cultured podocytes.

There is increasing evidence that polyunsaturated fatty acids (PUFA)-derived lipid mediators produced during the onset of the inflammatory response, such as lipoxin A4, resolvins and protectins, have potent biological actions in a variety of cell types *in vitro* and in many animal models of disease *in vivo* [25]–[27]. They have shown proresolving effects on inflammation [26]–[29] and salutary effects on diseases, such as lung diseases [30]–[34], ischemic-stroke [35] retinal degeneration [36], [37] and Alzheimer’s disease [38]. Lipoxins, RvE1 and RvD1 each modulate and reduce inflammatory pain behaviour [39]. RvE1 also abolishes TNF-alpha-evoked N-methyl-D-aspartic acid receptor hyperactivity in spinal dorsal horn neurons through inhibition of the extracellular signal-regulated kinase (ERK) signaling pathway [40]. In the present study, administration of RvD1 significantly reduced TNF-α-induced and ADR-induced loss of synaptopodin *in vitro* and *in vivo* and protected kidney function. The beneficial effects were associated with reduction of lysine acetylation in 14-3-3β and augmentation of interaction between 14-3-3β and synaptopodin, further extending the clinical potential of Resolvins in the treatment of chronic kidney diseases.

The 14-3-3 family of proteins interact with many other proteins to regulate their function. Thus, post-translocation modification of 14-3-3 proteins is critical for their functional role in many basic biologic processes [15]–17. In particular, 14-3-3β has been shown to bind to phosphorylated synaptopodin and thereby prevent synaptopodin degradation by cathepsin-L in podocytes [8]. Our demonstration that 14-3-3β undergoes a progressive increase in lysine acetylation and dissociates from synaptopodin is the first demonstration of this mechanism in a model of progressive glomerular disease. In addition, our study is the first demonstration that a member of the Resolvin family can modify 14-3-3 protein function. The direct demonstration that glutamine for lysine substitution, thereby mimicking acetylation, dramatically interrupts 14-3-3β/synaptopodin binding, provides strong evidence that one mechanism underpinning the protective effect of RvD1 treatment is through preventing 14-3-3β acetylation and consequent dissociation of the 14-3-3β/synaptopodin complex and synaptopodin degradation. Our findings are consistent with previous in vitro studies in which lysine to glutamine substitution abolished the interaction of 14-3-3β with a number of binding partner proteins [43]. Of note, RvD1 by itself had no acute effects on synaptopodin protein levels in podocytes, rather its acute effects to prevent TNF-α induced down-regulation of synaptopodin expression operated through modulation of the interaction of synaptopodin with 14-3-3β. At later time points, RvD1 also prevented TNF-α induced down-regulation of synaptopodin mRNA levels which may be important in the ability of RvD1 to prevent ongoing podocyte damage in ADR nephropathy.

RvD1 is known to act on human leukocytes via the ALX and GPR32 receptors [3], [44]. We identified ALX expression in mouse kidney and this was reduced in the later stages of ADR nephropathy which may have contributed to the lack of protection of late RvD1 treatment, although as we were unable to localise ALX expression to individual cell types, this remains speculative. In addition, we identified ALX expression by the mouse podocyte cell line and the ALX antagonist WRW^4^ abrogated the effect of RvD1 in these cells, arguing that the protection actions of RvD1 in podocytes likely operate via this receptor. The precise mechanism by which ALX activation affects 14-3-3β protein function and synaptopodin gene transcription is not known, although it is likely to be indirect. RvD1 activation of ALX may protect podocytes by inhibiting TNF-α receptor signalling. Liao et al [41] demonstrated that RvD1 can suppress LPS-induced IκBα degradation and NF-κB p65 nuclear translocation, a process which is central to the TNF-α induced signalling [42].

Tubulointerstitial fibrosis in ADR nephropathy, as in most types of progressive glomerulonephritis, is thought to be a secondary consequence of glomerular injury. The protection from interstitial fibrosis seen with early RvD1 treatment in which glomerular injury was suppressed is consistent with this notion. Interestingly, late RvD1 treatment did not affect the progression of interstitial fibrosis. This is different to our previous study in which RvE1 suppressed interstitial fibrosis in the obstructed kidney [19]. However, there is direct and irreversible tubular damage in the obstructed kidney model with glomeruli unaffected. These finding suggest that there may be important differences in underlying mechanisms driving interstitial fibrosis in the ADR model compared to the obstructed kidney.

A second possible mechanism for the protective effect of RvD1 in ADR nephropathy could be through modulation of the inflammatory response. Glomerular injury in this model is thought to be macrophage-dependent, and adoptive transfer of M2-type anti-inflammatory macrophages can suppress proteinuria and glomerulosclerosis [45]–[47]. Resolvins are known to induce an anti-inflammatory M2-type macrophage phenotype [48], [49]. In our study, early treatment with RvD1 not only decreased macrophage infiltration, but also promoted macrophage differentiation towards an M2 phenotype based on up-regulation of the M2 markers, arginase-1 and YM1. Interestingly, late RvD1 treatment did not affect either macrophage numbers or phenotype. Whether this lack of macrophage response to RvD1 treatment indicates a down-regulation of RvD1 receptor expression by macrophages or an inability to overcome established inflammation is unclear.

Late RvD1 treatment was unable to halt progression of glomerulosclerosis in ADR nephropathy. This is presumably due to substantial podocyte damage having already occurred in all glomeruli by day 14 following systemic ADR administration. This finding suggests that RvD1 is unable to intervene in established disease; however, it is important to remember that glomerular injury in human disease is a stochastic process occurring over months to years. Therefore, there may be potential for RvD1 treatment to protect unaffected or partially damaged glomeruli from developing marked podocyte damage and progressing to glomerulosclerosis.

In conclusion, this study provides the first description of a protective role for RvD1 treatment in experimental glomerular disease. This protective effect was primarily attributed to preventing acetylation of 14-3-3β and thereby inhibiting dissociation of the 14-3-3β/synaptopodin complex and thus stopping degradation and consequent down-regulation of synaptopodin in podocytes. RvD1 may provide a new therapeutic approach to halt the progression of chronic kidney disease.
